# Peripheral Arthritis and Tenosynovitis in a Patient With Brucellosis

**DOI:** 10.7759/cureus.35292

**Published:** 2023-02-22

**Authors:** Abdülbaki Elmas, Firdevs Ulutaş

**Affiliations:** 1 Department of Internal Medicine, Giresun University, Giresun, TUR; 2 Department of Rheumatology, Giresun University, Giresun, TUR

**Keywords:** inflammation, infections, tenosynovitis, arthritis, brucellosis

## Abstract

Brucellosis is an infectious disease caused by Gram-negative bacteria. Musculoskeletal system involvement is common in patients with brucellosis. Herein we report a patient who presented with bilateral arthralgia in the ankles and diffuse swelling of the left hand in the follow-up. He had been diagnosed with brucellosis and improved well after a 10-week duration of antibiotic therapy. Rheumatic diseases may have many infectious mimickers. Brucellosis is one of those that should be considered in the differential diagnosis.

## Introduction

Brucellosis is a zoonotic infectious disease caused by small, Gram-negative, non-encapsulated Coccobacilli. It leads to a granulomatous reaction. Osteoarticular involvement is common in approximately 10% to 85% of the patients. The most common sites are sacroiliac joints (up to 80%) and spinal joints (up to 54%) in the musculoskeletal system. Compared with spondylitis and spondylodiscitis, peripheral joints are rarely affected. Arthralgia, arthritis, bursitis, enthesopathy, osteomyelitis, and tenosynovitis can be seen alongside brucellosis [1]. Herein, we report a patient who presented with bilateral arthralgia in the ankles and diffuse swelling of the left hand. He had been diagnosed with brucellosis after serological markers and magnetic resonance imaging (MRI) findings.

## Case presentation

**Figure 1:**
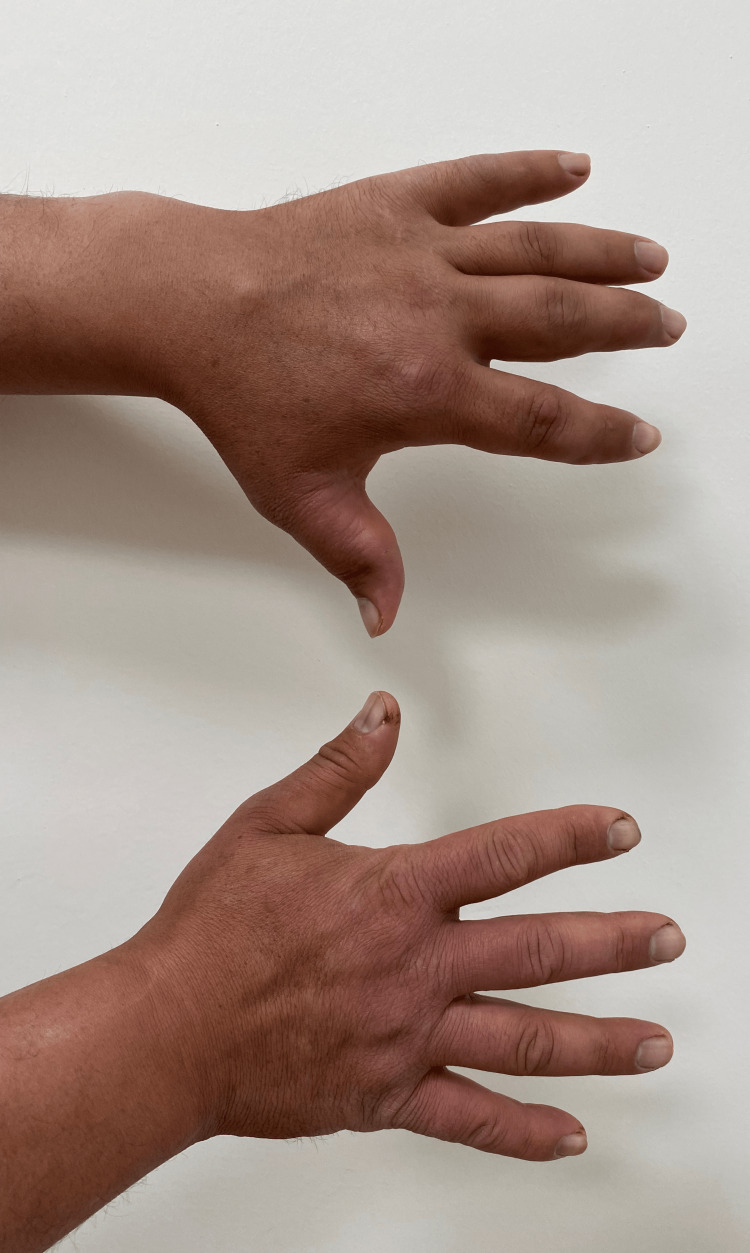
Isolated diffuse thickening of the left hand without any arthralgia.

A 37-year-old man diagnosed with arthritis of the bilateral ankles six months ago was treated as having reactive arthritis or undifferentiated arthritis. He had low-dose steroid treatment for about three months duration. Then, he was referred to the department of infectious diseases due to fever, joint pain, and diffuse left-hand swelling with a recent two-month history. With the Wright agglutination test, he was diagnosed with brucellosis and treated with rifampicin and doxycycline. In the fourth week of the treatment, he was referred to rheumatology due to an insufficient response. On physical examination of all systems, there was diffuse isolated thickening of the left hand without any arthralgia or other systemic involvement (Figure 1).

**Figure 2:**
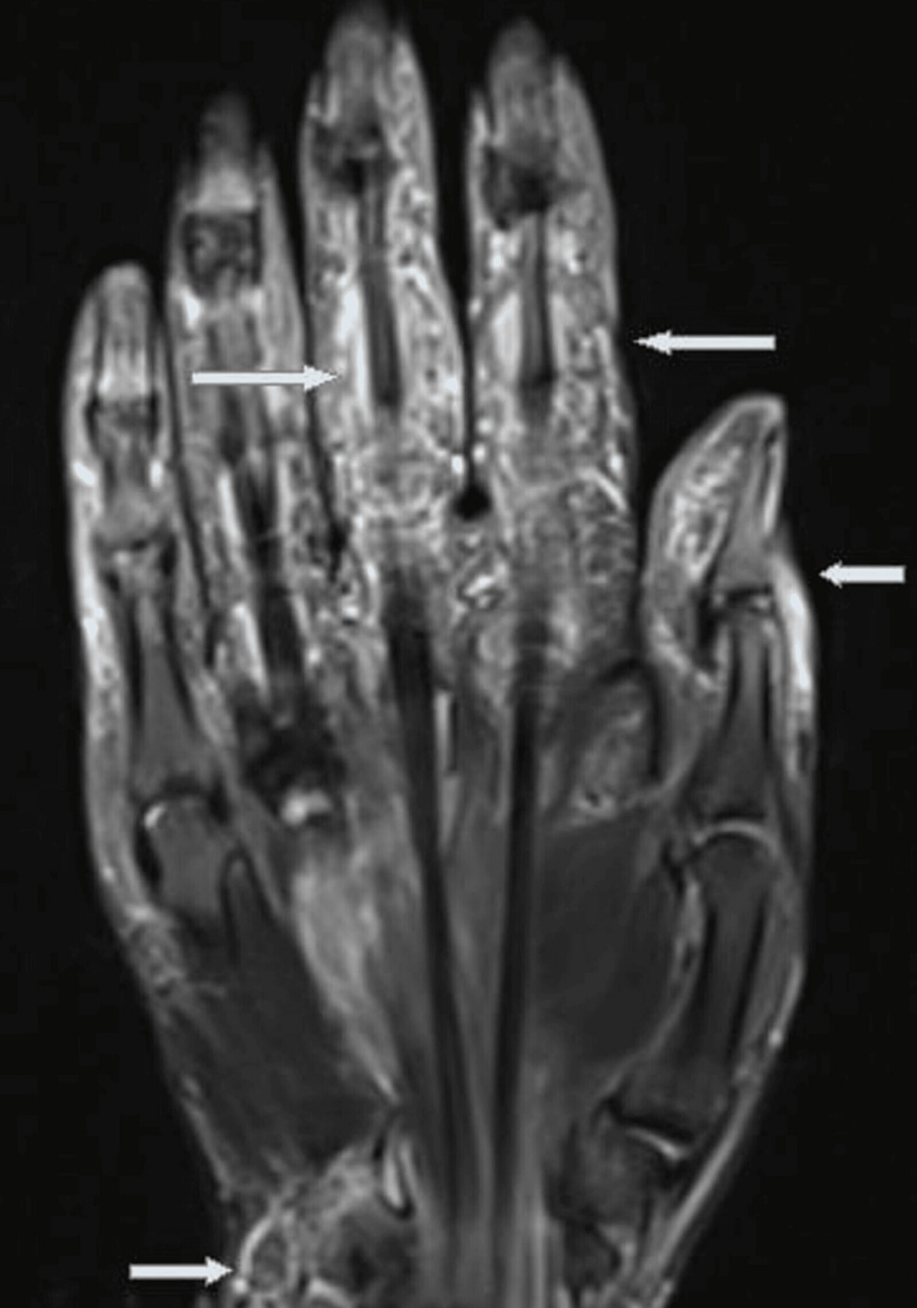
Tenosynovitis of the flexor tendon sheaths, synovitis of the intercarpal, and 2-3 metacarpophalangeal joints and medullary bone marrow edema of the capitatum were detected in the magnetic resonance imaging of the hand.

Radial and brachial pulses were precise. He also had no complaints about his other hand or ankles. Biochemical results were: negative acute phase reactants involving procalcitonin, erythrocyte sedimentation rate, and C-reactive protein; normal kidney; and live function tests. Serum uric acid, anti-cyclic citrullinated peptide (CCP) antibody, and rheumatoid factor were also negative. Arterial and venous doppler ultrasonography excluded peripheral arterial diseases or thrombosis. Tenosynovitis of the flexor tendon sheaths, synovitis of the intercarpal, two to three metacarpophalangeal joints, and medullary bone marrow edema of the capitatum were detected in the magnetic resonance imaging of the hand (Figure 2).

**Figure 3:**
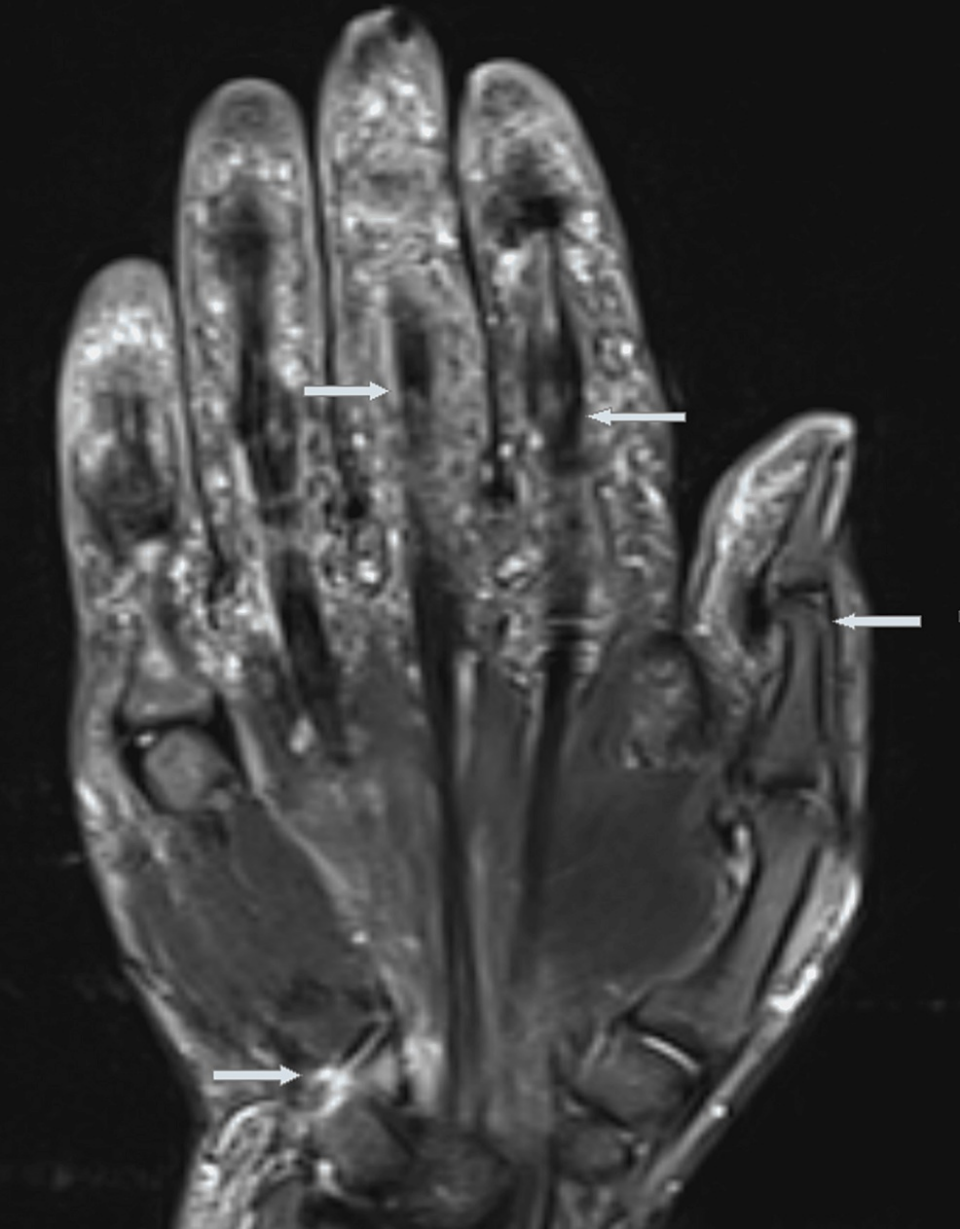
Improved MRI findings.

A diagnostic biopsy was performed to exclude mimicker diseases. Histopathological findings were related to a granulomatous inflammatory reaction accompanied by Langhans-type multinuclear giant cells. Further research investigations, including the serum QuantiFERON test, computed tomography of the lungs, serum angiotensin-converting enzyme (ACE) levels, and Bartonella henselae test for Cat-scratch disease, were negative for a definite diagnosis. The clinical signs did not match rheumatic diseases, and the patient's presentation was different. Also, it is well known that immunosuppressive drugs used to treat rheumatic diseases can aggravate underlying infections. So we used only antimicrobial agents, analgesics, and nonsteroidal anti-inflammatory drugs (NSAIDs) for his patient. We followed the patient with brucella-associated tenosynovitis. His arthritis had a self-limiting and chronic course and was responsive to treating the underlying cause. We extended the duration of the treatment to 10 weeks. His clinical symptoms and MRI findings improved well (Figure 3).

During his medical treatment, he also had physical therapy and muscle-strengthening exercises.

## Discussion

Many viral, bacterial, and/or parasitic infectious agents mimic rheumatic diseases such as Parvovirus B19, Chikungunya, Hepatitis B, Hepatitis C, human immunodeficiency virus (HIV), mycobacterial infections (Poncet's disease), Lyme disease, and brucellosis [2]. Brucellosis should be considered in the differential diagnosis of many rheumatological diseases, although tenosynovitis and peripheral arthritis are rare in these patients [3]. The patient is interesting in a few ways: the presentation of the patient was different from rheumatic diseases; there was diffuse hand swelling due to the common involvement of tendon sheaths and synovitis. The second point was that low-dose glucocorticoids lead to additional arthritic involvement.

Although there is no standard therapy for brucellosis, general guidelines are available, such as a combination of two or three antibiotics, long-term therapy to prevent relapses, and surgery for abscesses [4]. Batmaz et al. presented the case of Brucellosis with tenosynovitis of the extensor muscle of the arm. The patient recovered with a six-week treatment with doxycycline 200 mg/d and rifampicin 600 mg/d [5]. Lluch et al. reported a patient with flexor tenosynovitis in the hand. For ten weeks, their patient was treated with specific antibiotics. The authors emphasized macroscopic, rice-like bodies in the tendon's sheath [6].

Different osteoarticular manifestations require different treatment durations. For example, in studies, a triple regimen including streptomycin (1 g daily) plus doxycycline (100 mg twice daily) plus rifampin (15 mg/kg daily) for six months had 100% efficacy in brucella spondylitis [3]. Chronic, complicated brucellosis cases can need different treatment agents such as tigesiklin, trimethoprim-sulfamethoxazole, or fluoroquinolone [7]. It was emphasized that delayed treatment is associated with high relapse rates and treatment failure [8]. Magnetic resonance imaging is recommended to see the disease's extent and evaluate the effectiveness of treatment. In addition, MRI has important efficiency indisputably higher in soft tissue resolution [9]. Increased signals in the intervertebral disc and vertebral bodies on T2-weighted and contrast-enhanced sequences and soft tissue involvement are typical MRI findings of Brucella spondylitis [10]. However, it is not specific enough to be diagnostic [11].

## Conclusions

Rheumatic diseases can have many infectious mimickers. Therefore, steroids and disease-modifying anti-rheumatic drugs (DMARDs) are occasionally needed in different refractory and prolonged illnesses. Ruling out infectious diseases is very important before a clear diagnosis. Brucellosis infection should be considered in the differential diagnosis to prevent sequelae caused by tenosynovitis and arthritis.
